# Range expansion during recolonization: what does animal personality have to do with it?

**DOI:** 10.1093/beheco/araf053

**Published:** 2025-05-28

**Authors:** Radoslava Jánošíková, Filip Tulis, Ivan Baláž, Jana A Eccard, Valeria Mazza

**Affiliations:** Department of Environmental Sciences, Faculty of Natural Sciences and Informatics, Constantine the Philosopher University in Nitra, Tr. A. Hlinku 1, 949 74, Nitra, Slovak Republic; Department of Environmental Sciences, Faculty of Natural Sciences and Informatics, Constantine the Philosopher University in Nitra, Tr. A. Hlinku 1, 949 74, Nitra, Slovak Republic; Department of Environmental Sciences, Faculty of Natural Sciences and Informatics, Constantine the Philosopher University in Nitra, Tr. A. Hlinku 1, 949 74, Nitra, Slovak Republic; Animal Ecology, Institute for Biochemistry and Biology, University of Potsdam, Maulbeerallee 1, 14469, Potsdam, Germany; Animal Ecology, Institute for Biochemistry and Biology, University of Potsdam, Maulbeerallee 1, 14469, Potsdam, Germany; Department of Ecological and Biological Sciences, University of Tuscia, Largo dell’Università 1, 0100 Viterbo, Italy

**Keywords:** animal personality, information-gathering, spatial sorting, novel environments, range expansion, small mammals

## Abstract

At the edge of an ongoing expansion, pioneer individuals encounter novel ecological and evolutionary pressures that may not be experienced by conspecifics settled in long-colonized areas. Consistent behavioral differences among conspecifics (animal personality) may be important determinants of individuals’ successful colonization of novel environments and range expansion. By enhancing an individual’s ability to find food and shelter as well as increasing its capacity to navigate novel environments, behavioral traits such as exploration and risk-taking are thus expected to be more highly expressed in populations undergoing expansion than in established populations.

We investigated among-individual variation in behaviors associated to risk-taking and exploratory tendencies in populations of small mammals during different stages of the colonization process. Using a standardized behavioral test in the field, we quantified exploration and boldness of striped field mice (*Apodemus agrarius*, N = 95) from six subpopulations from Germany, where they are established, and in Slovakia, where a recolonization of the area is currently in progress, and in control species bank voles (*Myodes glareolus*, N = 76) that shared the same habitats but were long-established at all sites.

Striped field mice in the expanding populations were significantly slower in exploring the open field arena, while showing comparable levels of risk taking compared to conspecifics from established populations. No difference in behavior was detected between the populations of bank voles. Our results suggest that a slow exploration strategy might play an advantageous role in expansion processes of small mammal populations.

## Teaser

When animal populations expand into new areas, individuals face unique challenges which shape their behavior. This study examines the exploratory and risk-taking behaviors of striped field mice and bank voles during different colonization stages. We found that striped field mice at the expansion edge explored their surroundings more slowly, although they exhibited similar risk-taking compared to established conspecifics. Bank voles, long-established in all the same locations, did not show behavioral differences. These findings suggest a cautious exploration strategy may help small mammals to adapt to new environments during expansion.

## Introduction

Environmental alterations and climate change are drivers of range shift and distribution of species worldwide (eg Riley et al. 2017). Because the geographic ranges of most organisms are limited to some extent by climatic factors (eg Parmesan 2006), changes in global climate trends have caused many species to shift or expand their distributions to include altitudes or latitudes that provide more suitable conditions (eg Walther et al. 2002; Chen et al. 2011; Bellard et al. 2012; Riley et al. 2017), thus leading to radical alterations in natural ecosystems (eg Thomas et al. 2001; Hill et al. 2002; Riley et al. 2017). Such range expansions challenge animals to face a series of sequential, selective pressures (eg Blackburn et al. 2011; Chapple and Wong 2016) that can be conceptualized using the colonization pathway model, which involves four main ecological stages: arrival, establishment, increase, and spread (eg Evans et al. 2010; Sol et al. 2013; Rigg et al. 2025), and where each stage poses its own set of specific difficulties (eg Blackburn et al. 2011; Sol et al. 2013; Chapple and Wong 2016).

Effective and timely adjustments to such environmental barriers are often mediated by behavioral shifts (eg Atwell et al. 2012; Sol et al. 2013; Chapple and Wong 2016; Weis and Sol 2016; Lapiedra et al. 2017; Heger et al. 2019), because behavior entails reversible responses that allow individuals to shift within their repertoire (eg Wyles et al. 1983; Wcislo 1989; Huey et al. 2003; West-Eberhard 2003; Sol et al. 2005; Duckworth 2009; Camacho-Cervantes et al. 2015; Pereira et al. 2024). This “reactive” nature of behavior is what enables animals to actively interact with their environment (eg Duckworth 2009; Shettleworth 2009). Reversible responses enabled by this plasticity may not be feasible through slower processes such as adaptive changes in morphology or physiology (eg West-Eberhard 2003; Duckworth 2009) and therefore behavioral shifts play a crucial role in facilitating transitions through environmental barriers (eg Sol et al. 2013; Weis and Sol 2016; Ruland and Jeschke 2020). The degree to which animals can efficiently gather and use environmental information may then turn into a key component of survival and overall successful colonization of habitats (eg Sol et al. 2013; Haave-Audet 2024). At the edge of an ongoing expansion, pioneer individuals encounter ecological and evolutionary pressures that may not be experienced by conspecifics settled in long-colonized areas (eg Saul et al. 2013; Weis and Sol 2016; Gruber et al. 2018; Heger et al. 2019; Eccard et al. 2023, Mazza and Eccard 2024). This is because individuals at the expansion front cannot rely on prior experience or social information about the habitat they are spreading into, and still need to locate suitable shelter, food patches and mates, while facing local predators or interference by competing species (eg Saul et al. 2013; Chapple and Wong 2016; Weis and Sol 2016; Gruber et al. 2017; Heger et al. 2019; Eccard et al. 2023; Mazza and Eccard 2023). Therefore, the ecological challenges that pioneers meet are often best dealt with by specific behaviors such as a propensity to take risks, engage with novel stimuli, efficiently gather and store information for subsequent decision-making (eg Saul et al. 2013; Griffin et al. 2016, 2022; Weis and Sol 2016; Gruber et al. 2018; Heger et al. 2019; Eccard et al. 2023; Mazza and Eccard 2024; Mazza and Šlipogor 2024; Surkova et al. 2024). Conversely, different combinations of traits are predicted to be favored in areas where the population is already established, because of the costs of maintaining dispersal-promoting traits, or the presence of different selective pressures like enhanced intraspecific competition, resulting in a nonrandom distribution of behavioral phenotypes between expansion edge and established populations (eg Duckworth and Badyaev 2007; Duckworth 2008; Burton et al. 2010; Hudina et al. 2015; Canestrelli et al. 2016; Heger et al. 2019; Eccard et al. 2023; Mazza and Eccard 2024; Chuang and Riechert 2022; Chapple et al. 2022).

Despite being rapidly reversible, some behavioral responses can be highly stable in their level of expression (eg Réale et al. 2007; Duckworth 2009). Consistent within-species variation in behavior (ie animal personality; Dall et al. 2004; Sih et al. 2004, 2008; Bell et al. 2006; Réale et al. 2007) can be an important factor for dispersal success, a key in processes of range expansion (eg Duckworth 2008; Cote et al. 2010; Ducatez et al. 2012; Liebl and Martin 2012; Korsten et al. 2013), including in the context of biological invasions (eg Rehage et al. 2004; Fogarty et al. 2011; Chapple et al. 2012; Pyper et al. 2024). Animal personality is often described as between-individual differences in behavioral tendencies that are consistent across time and contexts (Réale et al. 2007). Thus, individuals often exhibit consistent differences in traits such as exploration, boldness, activity, sociability, and aggression as a response to their environmental conditions (Réale et al. 2007). Exploration is defined as the tendency to gather general information about the structural properties of the surroundings, without apparently serving any immediate need, a trait that has a crucial impact for animals’ direct needs such us foraging, mating, and dispersal (eg Réale et al. 2007; Mettke-Hofmann et al. 2009; Russell et al. 2010; Huang et al. 2016; Burstal et al. 2020). Boldness is defined as consistent among-individual variation in risk taking propensity (eg Réale et al. 2007) and illustrates how animals face the inherent challenges and risks entailed by the expansion into a novel habitat. These traits provide evidence for a behavioral polymorphism that may underlie long-term dispersal behavior (Fraser et al. 2001).

If the disperser’s personality type enhances its colonization success, then personality-dependent dispersal might play an important role in range expansions, and lead to spatial sorting, ie the accumulation of individuals with specific phenotypes at the expansion edge (eg Shine et al. 2011; Burstal et al. 2020; Galib et al. 2022; Eccard et al. 2023), where assortative mating may occur, thereby enhancing the expression of the traits favoring dispersal and colonization (eg Duckworth and Badyaev 2007; Duckworth 2008; Burton et al. 2010; Shine et al. 2011). Spatial sorting theory suggests that bolder individuals are more likely to colonize new habitats (Duckworth and Badyaev 2007; Shine et al. 2011), however, empirical findings are equivocal. Some studies from the expanding edge report bolder frogs and fish (e,g, Myles- Gonzales et al. 2015; Gruber et al. 2017) and shyer rodents (eg Hudina et al. 2014; Eccard et al. 2023), or no differences in boldness (eg Groen et al. 2012; Lopez et al. 2012), compared to the established range.

Additionally, high genetic relatedness among individuals at the edge promotes dispersal due to repeated founder effects (eg Fronhofer et al. 2015; Myles-Gonzalez et al. 2015; Gruber et al. 2017; Chuang and Riechert 2021). The general hypothesis is that with personality-dependent dispersal, individuals leading a range expansion might often display behavioral characteristics that facilitate the colonization of new habitats and hasten the colonization process (eg Cote et al. 2010; Canestrelli et al. 2016).

Studying ongoing processes of range expansion can give us insights into both the drivers of range expansions, as well as the role of behavior in dealing with novel environments under current global change. However, information on current range expansions is relatively scarce, and challenging to obtain because it requires identifying and following range expansions in progress. Moreover, most of currently monitored range expansions concern non-native species (eg Gruber et al. 2018; Burstal et al. 2020; Eccard et al. 2023; Mazza and Eccard 2024), leaving the expansions of native populations relatively uninvestigated (but see eg Thomas et al. 2001; Hill et al. 2002; Riley et al. 2017).

Here, we assessed the role of animal personality during the range expansion of striped field mice (*Apodemus agrarius*) through native central Europe. Striped field mice are small mammals widely distributed over the entire Palearctic region (Latinne et al. 2020), currently re-colonizing a former part of their range, which had been void of the species for over a century (Khlyap et al. 2021). The main objective of this study was to compare populations at different stages of the colonization process, with expanding populations representing the early phases, where individuals disperse into novel environments, and established populations reflecting later stages, where populations become locally adapted and stabilized over time. To understand how behavioral traits may differ across these stages, we examined dispersal-relevant behaviors, namely exploration and boldness between individuals of striped field mice at the edge of their current expansion (ie in a recently colonized area in Slovakia), with those of conspecifics from a long-established population in central Europe (ie in Eastern Germany). Animals with high exploratory tendencies are better equipped to discover resources and adapt to new environments, which can facilitate the colonization of previously unoccupied habitats (eg Cote et al. 2010; Liebl and Martin 2012; Burstal et al. 2020; Eccard et al. 2023). Bolder individuals are often found to disperse more and cover greater distances compared to those who are shyer (eg Cote et al. 2010; Myles-Gonzalez et al. 2015; Frynta et al. 2018). We therefore expected (a) nonrandom sorting for behavioral traits like exploration and boldness across expanding and established populations. We also expected that (b) exploration and boldness would be consistent across time and correlated with each other at the phenotypic level (eg Dammhahn et al. 2020), as both traits are known to influence risk-taking propensity and resource exploitation in novel environments (eg Sih et al. 2004; Réale et al. 2007; Carter et al. 2010; Bókony et al. 2012; Miranda et al. 2013; Ducatez et al. 2017; Lapiedra et al. 2017; Baxter-Gilbert et al. 2019). Such behavioral syndrome (Sih et al. 2004) is anticipated to enhance success at the expansion front (eg Bókony et al. 2012; Miranda et al. 2013; Ducatez et al. 2017; Lapiedra et al. 2017) and link both traits to dispersal and range expansion (eg Schirmer et al. 2019; Burstal et al. 2020; Bisconti et al. 2022). Since possible differences in behavioral responses of animals living in different places could also reflect adjustments to local habitat conditions or population structure (eg Réale et al. 2007; Stamps and Groothuis 2010), we also compared the same behavioral traits from populations of bank voles (*Myodes glareolus*), that share the study locations and have similar ecological requirements of striped field mice (eg Kozakiewicz 1987; Jancewicz and Gliwicz 2017; Schirmer et al. 2020) but are long-established both in Germany and Slovakia (eg Kornhuber 1857; Spitzenberger 1999). Thus, both species are equivalent functional types within an ecological community and occupy similar ecological niches (Schirmer et al. 2020). The bank voles in this study lived in the same locations, shared the same abiotic and biotic factors, experienced the same ecological conditions of the striped field mice, minus the expansion. If behavioral differences between individuals of different populations emerged in relation to range expansion, we expected that bank voles would show no behavioral differences between German and Slovakian populations. Alternatively, if potential differences between populations were driven by local adaptations, we expected individuals of both species to differ in a similar fashion.

## Methods

### Study species distribution over time

Striped field mice are well-established in some northern parts of central Europe (eg Germany) since the beginning of 19th century (Spitzenberger and Engelberger 2014; Petrosyan et al. 2023). However, in other regions, at the edge of their distribution (eg Czechia, Denmark), the species showed strongly fluctuating dynamics during last decades, and its spread into new territory was subsequently followed by a retreat from the colonized area over a period of several decades (eg Farský 1965; Polechová and Graciasová 2000; Bryja and Řehák 2002; Baagøe and Jensen 2007; Borkenhagen 2011; Heroldová et al. 2013).

The first occurrence of striped field mice was documented in the northern part of Slovakia at the end of the 19th century, based on trapping (eg Kocyan 1888 in Stanko 2012). Until the 1970s, the species was confirmed to occur only in eastern and northern parts of Slovakia (eg Dudich and Stollmann 1986; Dudich 1997). From 1980s the species began to spread westward to central parts of Slovakia. In 2010 the presence of striped field mice was confirmed in the southwestern part of Slovakia for the first time (Ambros et al. 2010), in an area where small mammal populations were rigorously monitored, and that had been devoid of prior occurrence of the species for more than a century (eg Méhely 1908; Balát 1956; Folk 1956; Pachinger et al. 1996, 1997;  Krištofík 1999, Noga 2009; Poláčiková 2010). Paleontological findings (Horáček, I. and Ložek 1993; Pazonyi et al. 2004; Obuch and Karaska 2010; Obuch et al. 2016; Obuch 2022) and parasitological research (Dudich 1997) indicated that this current spreading in part of central and eastern Europe represents a re-colonization of areas occupied by the species in the past, before a its retreat (Tulis et al. 2023). Since the first sighting in 2010, the progress of striped field mice expansion in south-western Slovakia is monitored regularly (Tulis et al. 2016), making the identification of the expansion edge relatively straightforward.

### Study design

We investigated among-individual differences in risk-taking and exploration between striped field mice at the expansion edge (in Slovakia) and in an established population (in Germany). To avoid potential confounds due to local conditions, we also assessed the same traits in bank voles, a species that shares the niche and ecological requirements of striped field mice (eg Kozakiewicz 1987; Jancewicz 2017; Schirmer et al. 2020) but is established in all sites (eg Kornhuber 1857; Spitzenberger 1999; Krištofík and Danko 2012). Bank voles represent the syntopically- and sympatrically occurring species for both striped field mice populations (eg Kornhuber 1857; Spitzenberger 1999). Both species colonized Europe during the Last Glacial period (eg Wójcik et al. 2010; Knitlová and Horáček 2017). Both species commonly co-occur in parts of central and eastern Europe in a variety of landscapes, including forests, agricultural landscapes, grasslands (eg Kozakiewicz 1987; Jancewicz 2017; Väli 2020), are similar in habitat choice (eg Kozakiewicz 1987; Kozakiewicz et al. 1999; Panzacchi et al. 2010; Fischer et al. 2011; Balestrieri et al. 2015; Jancewicz and Gliwicz 2017; Benedek and Sîrbu, 2018; Schirmer et al. 2020), diet (eg Gliwitcz 1981; Jancewicz and Gliwicz 2017), and have partially overlapping temporal activity patterns (eg Jancewicz and Gliwicz 2017). Both species are active during both day and night, however *Apodemus* mice can adjust their activity rhythms and phases in response to environmental factors, such as weather conditions (Wróbel and Bogdziewicz 2015) or habitat-related differences in predation pressure (eg Łopucki and Kiersztyn 2020). Also, our own observations confirm that striped field mice had activity periods distributed along the 24 h (Dammhahn et al. 2020), while voles are polyphasic with several independent short activity bouts (typically 2 to 3 h) and rest periods both during daytime and the night (eg Halle 2000; Lehmann and Halle 1987; Wolff 2008). Thus, on an individual level, animals are not active fully synchronously because while both species can be active at day and night, voles are polyphasic with short activity phases and long resting phases, and mice are flexible. Our approach employed the bank voles as a control against which the patterns from the comparison of the striped field mice populations could be set, helping to mitigate unobserved biases that may exist (Rosenbaum 1987; Krebs 1999).

Individuals who disperse into new territory are part of an expansion wave, the leading portion of which is the edge. The edge consists of the wave front and the demes immediately behind it (eg Chuang and Peterson 2016). Sites that were colonized during the last two years were thus considered as the striped field mice’s expansion edge. On the other hand, the populations settled and subsisted in Germany from the Holocene according to phylogenetic analyses, with records that indicate a stable presence of striped field mice in North-East Germany since the beginning of the 19^th^ century (eg Spitzenberger and Engelberger 2014; Latinne et al. 2020) were considered established populations.

### Study sites and trapping

**Fig. 1.**
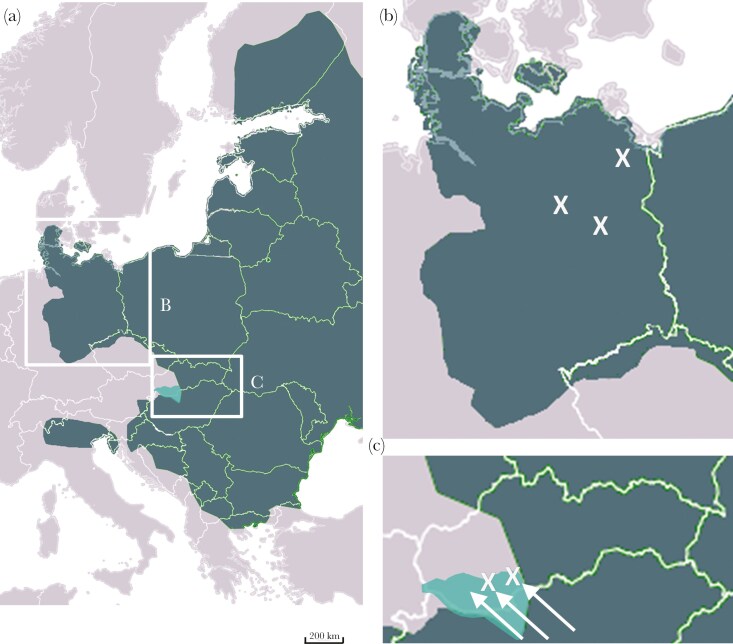
Distribution of *Apodemus agrarius* (shown in dark slate green) across Central and Eastern Europe (A, source: Wikipedia), including study regions in Germany (B) and Slovakia (C). Crosses indicate study sites, with direction of recent expansion of *Apodemus agrarius* in Slovakia from 2010 to 2024 (shown in turquoise- shaded area) marked by white arrows.

Data collection took place in six study sites in Slovakia (2) and Germany (4). In Slovakia, the study was conducted in rural fallow areas around the towns of Šintava (48.267629, 17.776484) and Lehota (48.305693, 17.973879) located in the northern Pannonian Plain and Danubian Highlands, adjacent to fallow land habitats. In Germany, study sites were located in the urban parks of Potsdam (52.403069, 13.025356) and the fallow lands of rural areas in northern (53.363195, 13.594960) and eastern Brandenburg (52°43’26.2, 12°15’01.9) (Figure 1) .

We captured animals using multiple-capture live traps (Ugglan Special Traps n. 1 to 2, Grahnab AB, Hillerstorp, Sweden). At each site, we used a capture-mark-recapture approach with 45 to 50 traps set in a regular grid of ca. 10 m distance between traps. Traps were pre-baited with oat flakes and apples for two nights, because pre-baiting live traps can significantly increase the trapping success of woodland rodents (eg Gurnell 1980). Traps were then activated between 18:00 and 19:00 h and checked and deactivated every morning between 06:00 and 07:00 h. Trapping was performed on each site continuously for 7 to 10 d. We ensured low bias towards trap-shy individuals by pre-baiting and continuing the trapping until the trapping success was < 2 novel individuals/grid night. The average time between behavioral tests (inter-trial interval) was 1.8 ± 1.6 d. Handling was conducted carefully, after the conclusion of behavioral tests, and limited to the initial capture, during which the animals were sexed, weighed, individually marked with unique fur markings, and measured for standard morphometric parameters. After the behavioral observations were concluded, all animals were released at the site of capture. No injuries or fatalities occurred during trapping, handling, or behavioral testing, as all procedures were designed to prioritize animal welfare and followed strict ethical guidelines. The study took place between July and October 2022.

### Boldness and exploration assessment

For behavioral testing, we adopted standard laboratory tests that are commonly used in personality studies of small mammals (eg Mazza et al. 2020; Schirmer et al. 2019-2020; Dammhan et al. 2020; Eccard et al. 2023;  Mazza and Eccard 2024) (exemplified in supplementary material video). The set-up combined the dark–light test (Young and Johnson 1991) and the open-field test (Archer 1973), and was adjusted to be executed under field conditions. Animals were tested directly in the field on the day of capture, with no prior handling before behavioral testing, precluding possible influences of handling stress on behavioral expression (eg Schirmer et al. 2019; Mazza et al. 2020). Animals were never handled before a behavioral test. ie on first capture they were phenotyped for behavior and afterwards marked and handled. On later captures, already marked animals were not handled at all. The dark–light test measures willingness of individuals to leave a dark and enclosed shelter to enter an unknown, bright and empty open field arena, which is perceived as potentially risky by rodents (eg Crawley and Goodwin 1980; Bourin and Hascoët 2003; Bloch and Belzung 2023). The open field test quantifies an individual’s exploratory activity in an unfamiliar space (eg Hall 1932; Gould et al. 2009). The test set-up consists of a dark plastic tube (10.5 cm Ø × 32 cm length) connected to a round PVC open field arena (130 cm Ø, 30 cm high). At the start of each test, animals were transferred without direct handling from the trap into a shuttle tube, which was then inserted into the set-up tunnel. The animal was left in the tube for 60 s for acclimatization. Afterwards we opened the door leading into the open field arena, and we measured the individual’s latency to emerge from the tunnel into the open field arena with its head (“latency to emerge’), as a proxy for risk-taking propensity. If the animals did not leave the dark tube within 5 min, they were gently guided into the open field arena by pushing the floor of the shuttle tube, and the latency was set to 300 s (0.19% of all tests). To reduce potential observer effects, we carried out observations from behind the emergence tube, so animals could not spot the observer before or while emerging. When the animal entered the circular open field arena, we closed the tube door and recorded the individual’s latency to reach the central part of the open field arena with its full body, excluding the tail (‘latency to OF center’), as a proxy for exploration tendency. If animals did not enter the central area within 5 min, the latency was set to the maximum of 300 s (0.06% of all tests). For those few instances where an animal did not exit within five minutes, it was gently guided into the open field arena, and any immediate escape movement following this was excluded from the analysis. Recaptured individuals were identified by their markings, re-tested using the same behavioral protocol, and released without undergoing repeated measurement procedures. Open field tests were video recorded with a camera (DigiProtect USB 120 Server, Version 6.212), to avoid potential disturbances from the researchers” immediate vicinity. After testing, animals were removed from the open field arena by offering their familiar trap as a hiding place, and the apparatus was cleaned with 70% alcohol. At the end of each test, the open field arenas were cleaned with 70% ethanol. All tests were conducted between 08.00 and 17.00 h, under natural light conditions. The open field arena was always set in the shade to avoid direct sunlight and shade patterns. It was not possible to record data blind because our study involved focal animals in the field. However, during video analyses, the observed (RJ) was blind to the identity of the individual.

### Statistical analyses

We investigated among-individual differences in risk-taking and exploration between striped field mice at the expansion edge in Slovakia and in an established control population in Germany. As a precondition to our comparison of individual behavior among established and expanding populations, we first tested whether individual behavioral responses were repeatable, ie consistent across time, and thus a useful representation of an animal’s behavioral type or personality (eg Réale et al. 2007). Repeatability is the proportion of phenotypic variation that can be attributed to between-individual variation relative to the variation in the population (eg Nakagawa and Schielzeth 2010; Dingemanse and Dochtermann 2013). We calculated repeatabilities for the latency to emerge from the dark-light tube into the open field area (as a proxy for risk-taking propensity), and the latency to enter the central part of the open field arena (as a proxy for exploration strategy) for all tested animals. We used the *rptR* package (Nakagawa and Schielzeth 2010; Stoffel et al. 2017), with individual ID as a random factor, and estimated 95% confidence intervals (CI) of repeatabilities for each variable by parametric bootstrapping (N = 1000 simulation iterations) and P values by 1000 permutations. Latencies were log-transformed to meet the normality assumption.

We then used restricted maximum-likelihood linear mixed effects models (LMMs) with a Gaussian error distribution to evaluate inter-individual differences in behavior between rodents in expanding and established populations. We included region (Germany vs Slovakia), and round of testing as fixed factors in all models. Sex and age class were initially included as fixed factors and excluded from final models based on their lack of explanatory importance using stepwise backward model selection based on log-likelihood ratio tests comparing nested models (Zuur et al. 2009). Interactions between explanatory variables were initially included, then dropped when non-significant. To account for repeated measures, we added the individual identity as a random intercept nested in trapping/testing site. We ensured that there was no strong collinearity between model predictor variables (ie Spearman’s rank correlation coefficient > 0.70) before analyses. We ran separate sets of models for striped field mice and bank voles.

Since some of the study sites in Germany were found in green urban plots, we ran a Mann-Whitney-U test to compare the type of surrounding matrix (rural vs urban) within the German subset of data, to check for possible differences due to anthropogenic disturbance. We used the R package *lme4* (Bates et al. 2015). Visual inspection of residual plots did not reveal any obvious deviation from homoscedasticity or normality. The accepted significance level was ≤ 0.05. All data analyses were conducted with R version 3.2.3 (R core team, 2015).

### Ethical note

Experiments were performed in accordance with all applicable international, national and/or institutional guidelines for the use of animals, including the ASAB/ ABS guidelines for the Use of Animals in Research. Animal capture and behavioral tests were conducted under the permission of the “Landesamt für Umwelt, Gesundheit und Verbraucherschutz Brandenburg” (reference numberV3-2347-7-2019) in Germany and the Ministry of Environment of the Slovak Republic (reference number: 4850/2019–6.3).

We took great care in ensuring the animals’ welfare throughout the experimental procedure. Trapping was conducted using multiple-capture live-traps (Ugglan Special Traps n. 1 to 2, Grahnab AB, Hillerstorp, Sweden), equipped with plenty of food and apples as water sources. Hay provided thermal insulation and the opportunity to build a nest. We covered the traps with grass, branches, and leaves to provide shade and avoid overheating. In case of rainy nights, trapping was suspended. Traps were also weighted down to avoid disturbance by nocturnal predators (eg raccoons or foxes), but in one instance (in Germany) 9 animals were killed during the night, and we suspended the trapping immediately, leaving the site altogether. No injuries or fatalities occurred due to handling and behavioral testing in any site. Occupied traps were refilled with food and water sources and kept in the shade until testing. Lactating females were immediately released at the site of capture. Handling was done with care and only on first capture. Individual markings were based on noninvasive fur cutting. Animals were released at the capture site immediately after testing.

## Results

We quantified risk taking propensity and exploration of 95 striped field mice (38 in the established and 57 in the expanding population) and 76 bank voles (55 in Germany and 21 in Slovakia), using personality tests established for behavioral phenotyping of small mammals under field conditions (eg Mazza et al. 2020). A total of 243 tests were performed, and 70 individuals could be tested multiple times (average tests per individual: 1.6 ± 0.6 SD) because they were captured multiple times.

**Fig. 2.**
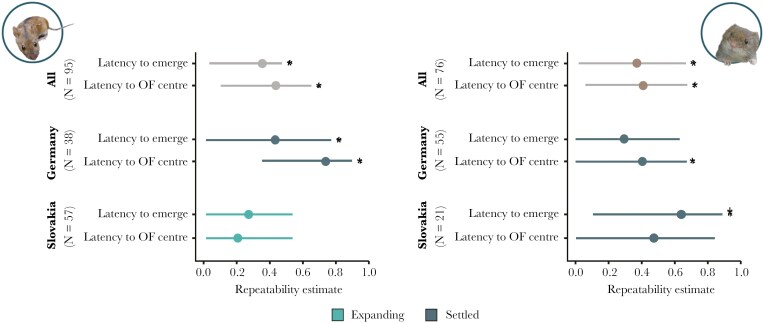
Repeatability estimates (R) and their 95% confidence intervals of behavioral variables for 95 individual striped field mice (*Apodemus agrarius*) (left) and 76 individual bank voles (*Myodes glareolus*) (right) quantified in repeated short behavioral tests, combined and separated for region of origin. Shown are means and 95% credibility intervals.

At dataset level, ie across all regions and expansion stages, both behavioral responses were repeatable over time in both species (Fig. 2 and Table S1 in the Supporting information). While region-specific repeatabilities were based on smaller sample sizes, they suggested that behavioral traits might be more repeatable in established than expanding striped field mice populations, while no such pattern emerged for bank voles (Fig. 2 and Table S2 in the Supporting information). Latency to emerge from the shelter and latency to reach the center of the open field arena were moderately correlated at the phenotypic level both in striped field mice (r_s_ = 0.28; *P* < 0.001) and bank voles (r_s_ = 0.35; *P* < 0.001).

**Fig. 3.**
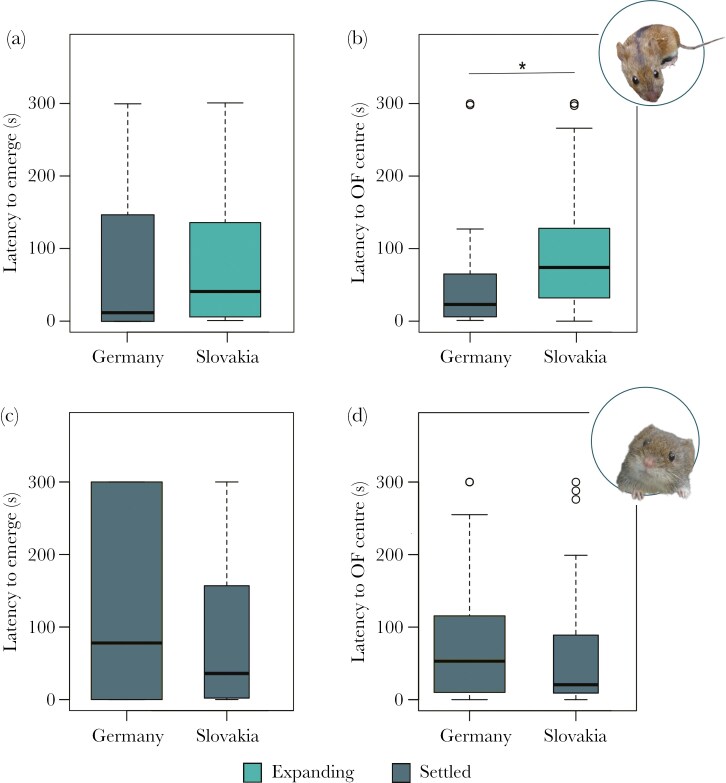
Latency to emerge from the dark shelter into the open field arena (a, c) and latency to explore the central part of the open field arena (b, d) for 95 individual striped field mice (*Apodemus agrarius*) (a, b) in the settled and in the expanding population, and 76 individual bank voles (*Myodes glareolus*) (c, d) in same sites of the two study regions Germany, where all animals were long-settled, and Slovakia, where striped field mice were expanding and bank voles were long-settled. Asterisk indicates significant differences from zero with *P* < 0.05, width of box indicates relative sample size. Data from all 243 tests is represented.

Striped field mice undergoing a range expansion (Slovakia) had a longer latency to explore the central part of the open field arena (*β *= 0.94 ± 0.25, *z* = 3.74, *P* < 0.001—Fig. 3b; Table S2) compared to conspecifics in established populations (Germany). However, animals from both populations were equally prone to emerge from the dark shelter (*β *= 1.06 ± 0.56, *z* = 1.89, *P* = 0.06—Fig. 3a; Table S2). Latency to explore the central part of the open field arena for all mice increased with testing round (*β *= 0.44 ± 0.20, *z* = 2.24, *P* = 0.025—Table S2). Bank voles tested in the same sites as the striped field mice showed no difference between regions in either the latency to emerge from the shelter (*β *= -0.14 ± 0.24, *z* = 0.59, *P* = 0.56—Fig. 3c; Table S2), nor in the latency to explore the center of the open field arena (*β *= -0.69 ± 0.57, *z* = -1.22, *P* = 0.22—Fig. 3d; Table S2). The type of habitat (rural/urban) surrounding the trapping sites had no effect on any behavioral variable in either species (striped field mice—latency to emerge: W = 295.5, *P* = 0.50; latency to OF center: W = 272, *P* = 0.87; bank voles—latency to emerge: W = 409, *P* = 0.13; latency to OF center: W = 567, *P* = 0.67).

Discussion

We found evidence of nonrandom sorting for exploratory behavior of individuals between expansion edge and established populations of striped field mice, while settled and expanding mice were similar in terms of risk-taking propensity. Bank vole individuals that came from the same sites as the striped field mice, but in both regions belonged to long-established populations, showed no differences in either exploration or boldness.

As expected, behavioral variables were repeatable across repeated tests in both species, supporting the reliability of these behavioral traits as indicators of personality (eg Bell et al. 2009). Repeatability estimates represent the upper boundary of heritability and genetic influences (eg Lessells and Boag 1987; Falconer and McKay 1996) and are considered a valid tool for making inferences on the evolutionary pressures shaping such variation (eg Dochtermann et al. 2015; Fronhofer and Altermatt 2017). We used the latency to enter the center of the open field arena as a proxy of exploration strategy, defined as the willingness or reluctance to gather environmental information. This latency reflects the speed and depth involved in information acquisition, which can vary with individual behavioral type (eg Sih and Del Giudice 2012). In line with our hypothesis, we found that striped field mice at the expansion edge differed in the way they approached the exploration of a novel space from conspecifics in long-established populations. Mice from expanding populations were indeed slower explorers, which contradicts the common assumption that pioneering individuals in expanding populations typically employ a fast exploration strategy (eg Burstal et al. 2020). Exploration strategies can reflect the different demands posed by the specific stages of the expansion process. These stages, arrival, establishment, increase, and spread (Evans et al. 2010), pose distinct behavioral challenges, particularly at the expansion front where individuals face heightened uncertainty. Different behavioral phenotypes may then be favored in different stages of the colonization process based on the novelty and (un)familiarity of the environment (habitat-dependent hypothesis, Réale et al. 2007). Expanding populations require first and foremost an accurate mapping of new territory, its dangers and resources, and pioneer individuals face this challenge in absence of conspecific cues, whereas in settled, long-established populations, environmental information can also be gathered from conspecifics and may thus require faster and potentially less accurate strategies (eg Heger et al. 2019; Mazza and Eccard 2024). Longer latencies to enter unfamiliar parts of the open field arena suggest that striped field mice at the expansion edge could have acquired more information about their surroundings before moving into new/unfamiliar ones. A slow strategy of information acquisition may be favored when expanding into an area that is void of conspecifics, and for which more or more detailed environmental information is needed (eg Hall and Kramer 2008; Carvalho et al. 2013). The coping style theory posits that slow and accurate explorers are more sensitive to changes in environmental cues, while fast explorers tend to rely on routines formed on shallow sampling (eg Benus et al. 1990; Verbeek et al. 1994; Koolhaas et al. 1999, 2010; Coppens et al. 2010; Sih and Del Giudice 2012). Slow exploring individuals may then fare better in unfamiliar or unpredictable environments, because their slower pace of exploration could make them more attuned and adaptable to changes in their surroundings, while fast explorers are expected to be favoured in a familiar, predictable environment, where they can form stable behavioral routines (eg Carere et al. 2010; Guillette et al. 2011; Šlipogor et al. 2022).

Our findings in relation to the differences between settled and expanding populations—with expanding populations taking longer to explore the center of the open field arena—align with patterns observed in previous studies reporting spatial sorting for behavioral traits between expansion edge and settled populations (eg Gruber et al. 2017, 2018; Bensky and Bell 2020; Burstal et al. 2020; Magory Cohen et al. 2020; McCune et al. 2020; Bisconti et al. 2022). Specifically, some studies reported slower exploration strategies, and a lack of strong correlation between boldness and dispersal behavior during the rapid expansion of jewelfish (*Hemichromis letourneuxi*) (Lopez et al. 2012), sun fish (*Lepomis gibbosus*) (Ashenden et al. 2017), and bank voles (Eccard et al. 2023; Mazza and Eccard 2024). Although facing similar challenges of unpredictable, novel environments, pioneer striped field mice in this study had not developed bolder phenotypes, as it was shown earlier for forest rodents in urban environments (eg Dammhahn et al. 2020; Mazza et al. 2020) compared to their rural reference populations; further indicating the different nature of urbanization and range expansion processes (eg Mazza and Šlipogor 2024). In the present study we also did not detect behavioral differences between individuals originating from settled populations in rural and urban sites. This can be in part due to the limited sample size from the urban area, but it is also possible that the urban parks where we conducted the data collection did not present typical urban-related challenges to the animals, that thus did not have to adjust their behavior in any way. In fact, previous studies showed that the degree of urbanization scales positively with the levels of exploration and risk-taking expressed by striped field mice (eg Dammhahn et al. 2020) and other small mammals (eg Mazza et al. 2020), so it is possible that the comparatively small town of Potsdam and its extensive parks were simply not “urbanized” enough to elicit behavioral adjustments.

Previous studies on pioneer small mammals expanding their range in non-native areas showed a marked adjustment of risk-taking levels, and specifically that individuals at the expansion edge were more risk-averse compared to settled conspecifics (Eccard et al. 2023). So our current findings do not align with this previously described pattern, which could be due to (i) species-specific idiosyncrasies, as previous studies referred to other species, and/or to (ii) risk-taking that could be strongly conserved in striped field mice (eg Mazza et al. 2021). Alternatively (iii), the different patterns regarding boldness could be due to the native vs non-native status of the area the range expansion takes place in. If the latter, it is possible that native striped field mice are pre-adapted to face the risks of these new territories they are colonizing, having inhabited these areas during part of the species’ evolutionary history. While striped field mice have relatively short generation times (typically 1 to 3 generations per year) (Baláž et al. 2013) behavioral traits such as risk-taking propensity could be maintained for several generations, especially if they are beneficial across a range of ecological conditions. Moreover, risk-taking behavior may be strongly conserved in this species, as suggested by previous findings (eg Mazza et al. 2021 for urban environments), and thus not prone to rapid shifts in response to range dynamics. Further studies should address this possibility, with explicit empirical testing of responses to familiar and unfamiliar dangers such us predators or competitors during range expansions in native and non-native habitats. This becomes of high interest in light of the expected climate-driven range shifts of several native species (eg Walther et al. 2002), and the level of preparedness they may require, to face the challenges and risks entailed by dispersal and range expansion.

Differences in the speed of climate-driven range shifts can lead to spatial mismatches between previously interacting species, thereby creating unique community assemblages and inter-species interactions (eg Le Roux and McGeoch 2008; Lurgi et al. 2012). The arrival of striped field mice has implications in relation to zoonoses transmission (eg Kraljik et al. 2016) and can alter the complex dynamics in the small mammal community composition (eg Tulis et al. 2023). However, bank voles in our study did not show any signs of behavioral adjustment in response to the recent reappearance of striped field mice and expressed similar levels of exploration and boldness compared to their conspecifics in Germany that have been coexisting with striped field mice for decades (eg Spitzenberger 1999; Spitzenberger and Engelberg 2014). In both regions mice and voles are partially segregated in their daily activity patterns, which could decrease strong behavioral interference (eg Schirmer et al. 2020).

Besides the reappearance of striped field mice in the community, bank voles in the Slovakian sites belong to settled, long-established populations, comparable to those tested in Germany. Populations of the same species can differ substantially in several aspects of their behavior, and these differences often depend on a multitude of biotic and abiotic factors (eg Frost et al. 2013; Sommer-Trembo et al. 2016; Mazza et al. 2020;  Bisconti et al. 2022). This is why we chose to collect data from a species that occupies highly similar ecological niche, co-occurs at the same locations, thereby being exposed to comparable abiotic and biotic factors, experiences the ecological conditions of the species of interest but not undergoing range expansion. We thus controlled both for within-species differences comparing expanding and settled populations as well as within-site micro-habitat effects due to local conditions. Our results on voles strengthen the results of parallel tests on mice, suggesting that environmental or temporal variables (eg Réale et al. 2007; Stamps and Groothuis 2010), which would likely have affected both species, are unlikely to be the main drivers of the observed behavioral differences. Instead, our findings point to expansion as a likely contributing factor underlying this variation, rather than influences such as predation pressure, weather conditions, or microhabitat differences.

In conclusion, our study shows that expanding and settled striped field mice differ in the strategies adopted to gather environmental information. These different strategies appear to represent adaptive adjustments for coping with range expansion, a process that requires careful mapping of novel surroundings and heightened sensitivity to changes (eg Koolhaas et al. 1999; Carere and LoCurto 2011; Sih and Del Giudice 2012). Our results suggest that in some behavioral traits, native species expanding their range (the striped mouse in this study) may exhibit parallels to invasive species introduced into non-native areas (slower level of exploring) but in others respects (difference in risk-taking levels between populations on expansion edge and established population) significant divergences may be evident.

These findings highlight the importance of considering species’ ecology when formulating predictions about the determinants of successful range expansions and biological invasions. Our results align with the broader patterns observed across species (eg Lopez et al. 2012; Ashenden et al. 2017; Eccard et al. 2023; Mazza and Eccard 2024), indicating that behavioral strategies are intricately linked to dispersal and colonization dynamics of new territories. The current ongoing expansion of striped field mice highlight the complex interplay between environmental changes and exploratory behavior, illustrating how intrinsic behavioral characteristics (animal personality) contribute to the successful colonization and re-colonization of new areas. This underscores the importance of information gathering in adaptive strategies for species facing dynamic and challenging environments.

## Supplementary Material

araf053_suppl_Supplementary_Data

araf053_suppl_Supplementary_Tables_S1-S2

araf053_suppl_Supplementary_Tables_S3

## Data Availability

Analyses reported in this article can be reproduced using the data provided by Jánošíková et al. (2025). Analyses reported in this article can be reproduced using the data provided in table S3 in the Supplements.
