# Supplementary material for: Range expansion during recolonization: what does animal personality have to do with it?
Source: Behav Ecol. 2025 May 28;36(4):araf053. doi: 10.1093/beheco/araf053 (PMC12168446; doi:10.1093/beheco/araf053)
Supplement: araf053_suppl_Supplementary_Tables_S1-S2 [file araf053_suppl_supplementary_tables_s1-s2.doc]

**Supplementary material**

|  |  | **All** | | | | |  | **Germany** | | | | |  | **Slovakia** | | | | |
| --- | --- | --- | --- | --- | --- | --- | --- | --- | --- | --- | --- | --- | --- | --- | --- | --- | --- | --- |
|  |  | R | SE | CI | | P |  | R | SE | CI | | P |  | R | SE | CI | | P |
| **SFM** | Latency to emerge | **0.36** | 0.13 | 0.08 | 0.59 | **0.011** |  | **0.42** | 0.23 | 0 | 0.79 | **0.045** |  | 0.26 | 0.15 | 0 | 0.56 | 0.08 |
|  | Latency centre | **0.42** | 0.12 | 0.14 | 0.61 | **0.003** |  | **0.73** | 0.14 | 0.41 | 0.91 | **0.001** |  | 0.20 | 0.15 | 0 | 0.50 | 0.16 |
| **BV** | Latency to emerge | **0.39** | 0.16 | 0.03 | 0.63 | **0.021** |  | 0.29 | 0.18 | 0 | 0.63 | 0.104 |  | **0.65** | 0.19 | 0.1 | 0.89 | **0.02** |
|  | Latency centre | **0.40** | 0.15 | 0.07 | 0.66 | **0.018** |  | **0.39** | 0.18 | 0 | 0.69 | **0.035** |  | 0.47 | 0.24 | 0 | 0.84 | 0.09 |

TABLE S1 - Repeatability estimates (R), corresponding standard error (SE), and 95% confidence intervals across region of origin (Germany vs Slovakia) for 95 individual striped field mice - SFM (*Apodemus agrarius*) and 76 individual bank voles - BV (*Myodes glareolus*), combined and separated for region of origin. For statistically significant effects P-values are highlighted in bold font.

|  | **Variable** | **Distribution** | **Estimate** | **SE** | **z** | **P** | **R^2^_m_** | **R^2^_c_** |
| --- | --- | --- | --- | --- | --- | --- | --- | --- |
| *Striped field mice* | *Latency to emerge* |  |  |  |  |  |  |  |
|  | Intercept | Gaussian (log) | 3.93 | 0.70 | 5.63 | < 0.001 | 0.03 | 0.36 |
|  | Region (Slovakia) |  | 1.06 | 0.56 | 1.89 | 0.059 |  |  |
|  | Test round |  | -0.09 | 0.44 | -0.21 | 0.835 |  |  |
|  | *Latency OF centre* |  |  |  |  |  |  |  |
|  | Intercept | Gaussian (log) | 2.48 | 0.31 | 7.93 | < 0.001 | 0.14 | 0.42 |
|  | **Region (Slovakia)** |  | 0.94 | 0.25 | 3.74 | **< 0.001** |  |  |
|  | **Test round** |  | 0.44 | 0.20 | 2.24 | **0.025** |  |  |
| *Bank voles* | *Latency to emerge* |  |  |  |  |  |  |  |
|  | Intercept | Gaussian (log) | 1.80 | 0.25 | 7.31 | < 0.001 | 0.02 | 0.44 |
|  | Region (Slovakia) |  | -0.14 | 0.24 | -0.59 | 0.557 |  |  |
|  | Test round |  | -0.26 | 0.17 | -1.57 | 0.117 |  |  |
|  | *Latency OF centre* |  |  |  |  |  |  |  |
|  | Intercept | Gaussian (log) | 5.30 | 0.58 | 9.15 | < 0.001 | 0.02 | 0.40 |
|  | Region (Slovakia) |  | -0.69 | 0.57 | -1.22 | 0.222 |  |  |
|  | Test round |  | -0.12 | 0.40 | -0.30 | 0.767 |  |  |

TABLE S2 - Model results for latency to emerge (in seconds) from the dark shelter and exploration activity in relation to region (Germany vs Slovakia), and test round for 95 individual striped field mice (*Apodemus agrarius*) and 76 individual bank voles (*Myodes glareolus*). R_m_ reports the marginal R² value based on the fixed factors, R_c_ the conditional R² value including the study site as a random factor. Reference levels are given in (). For statistically significant effects P-values are highlighted in bold font.
